# A novel high performance in-silico screened metagenome-derived alkali-thermostable endo-β-1,4-glucanase for lignocellulosic biomass hydrolysis in the harsh conditions

**DOI:** 10.1186/s12896-020-00647-6

**Published:** 2020-10-19

**Authors:** Shohreh Ariaeenejad, Atefeh Sheykh Abdollahzadeh Mamaghani, Morteza Maleki, Kaveh Kavousi, Mehdi Foroozandeh Shahraki, Ghasem Hosseini Salekdeh

**Affiliations:** 1 Department of Systems and Synthetic Biology, Agricultural Biotechnology Research Institute of Iran (ABRII), Agricultural Research Education and Extension Organization (AREEO), P. O. Box: 31535-1897, Karaj, Iran; 2 Laboratory of Complex Biological Systems and Bioinformatics (CBB), Institute of Biochemistry and Biophysics (IBB), University of Tehran, Tehran, Iran; 3 Department of Molecular Sciences, Macquarie University, Sydney, NSW Australia

**Keywords:** Novel endo-β-1,4-glucanase, In-silico screening, Metagenome, Enzymatic hydrolysis, Rice straw

## Abstract

**Background:**

Lignocellulosic biomass, is a great resource for the production of bio-energy and bio-based material since it is largely abundant, inexpensive and renewable. The requirement of new energy sources has led to a wide search for novel effective enzymes to improve the exploitation of lignocellulose, among which the importance of thermostable and halotolerant cellulase enzymes with high pH performance is significant.

**Results:**

The primary aim of this study was to discover a novel alkali-thermostable endo-β-1,4-glucanase from the sheep rumen metagenome. At first, the multi-step in-silico screening approach was utilized to find primary candidate enzymes with superior properties. Among the computationally selected candidates, PersiCel4 was found and subjected to cloning, expression, and purification followed by functional and structural characterization. The enzymes’ kinetic parameters, including V_max_, K_m_, and specific activity, were calculated. The PersiCel4 demonstrated its optimum activity at pH 8.5 and a temperature of 85 °C and was able to retain more than 70% of its activity after 150 h of storage at 85 °C. Furthermore, this enzyme was able to maintain its catalytic activity in the presence of different concentrations of NaCl and several metal ions contains Mg^2+^, Mn^2+^, Cu^2+^, Fe^2+^ and Ca^2+^. Our results showed that treatment with MnCl_2_ could enhance the enzyme’s activity by 78%. PersiCel4 was ultimately used for enzymatic hydrolysis of autoclave pretreated rice straw, the most abundant agricultural waste with rich cellulose content. In autoclave treated rice straw, enzymatic hydrolysis with the PersiCel4 increased the release of reducing sugar up to 260% after 72 h in the harsh condition (T = 85 °C, pH = 8.5).

**Conclusion:**

Considering the urgent demand for stable cellulases that are operational on extreme temperature and pH conditions and due to several proposed distinctive characteristics of PersiCel4, it can be used in the harsh condition for bioconversion of lignocellulosic biomass.

## Background

Lignocellulose is the principal constituent of the biomass and is the most abundant bio-renewable organic resource which is composed of three biopolymers, hemicellulose, cellulose and lignin [1]. The rice straw, wheat straw, corn straw, and sugarcane bagasse are the chief available agricultural wastes [2] and among them, rice straw (RS) has the most abundance over the world, However, only a small portion of this great resource is being used as animal feed, and the rest is squandered commonly by burning, which contaminates the air and causes various environmental perils [3, 4].

Naturally, complete hydrolysis of cellulose is achieved through the cooperative action of enzymes including cellobiohydrolase (EC 3.2.1.91), endo-β-1,4-glucanase (EC 3.2.1.4) and β-glucosidase (EC 3.2.1.21) [5]. Among the three types of cellulose-degrading enzymes, endo-β-1,4-glucanase is the crucial enzyme that first acts on the cellulose polymers’ amorphous sites and randomly cleaves the internal β-1,4-bonds and releases smaller fragments of varying random lengths [6]. Due to the sequence similarities of the catalytic domains of endo-β-1,4-glucanase enzymes were classified into glycoside hydrolase (GH) families of 5, 6, 7, 8, 9, 12, 44, 45, 48, 51 and 74 [7, 8].

Alongside with the vast involvement and importance in bio-ethanol production technologies, endo-β-1,4-glucanases are also applicable in various other industries such as pulp and paper, textile, food and feed processing, detergents [9].

The bioconversion process of lignocellulosic biomass consists of three steps, namely pretreatment of biomass, enzymatic hydrolysis of the polysaccharides, and fermentation of fermentable sugars. The densely packed structure of cellulose and hemicellulose together with lignin, which essentially serves as a protection for plants, highlights the importance of pretreatment strategies to solubilize and separate one or more of these components of biomass in order to make the remaining solid matter more easily accessible for the enzymatic hydrolysis [10, 11]. Several pretreatment methods have been designed and used all of which attempt to accomplish main purposes including increasing the digestibility of solid biomass to augment the sugar yield during enzymatic hydrolysis step, evading the degradation of released sugars, and minimize the risk of emergence of inhibitors which could potentially interfere with subsequent steps [12]. The biomass pretreatment often takes place in an acidic or alkaline and at high temperatures and the neutralization of these acids or bases generates salts [13]. The process of removing the formed salts before further downstream steps requires tons of water and energy; hence, halotolerant and halophile enzymes that are stable and operational in the presence of salts are in great demand [9, 14]. Numerous studies introduced novel cellulases with unique attributes, making them suitable for particular applications [15]. Some of the endo- β-1,4-glucanases were directly isolated from microorganisms. For instance, a new thermostable and halotolerant endoglucanase was produced from *Botrytis ricini* URM 5627 [16]. In another research, a thermo-halotolerant and alkali stable GH6 endoglucanase from *T. halotolerans* were discovered that displays high catalytic activity in alkaline pH conditions and the presence of NaCl. Furthermore, pretreatment with high concentrations of NaCl improved the enzyme’s activity [17]. A novel GH5 cellulase from *Aspergillus glaucus* with high stability under heat, acid, alkaline and saline conditions and ability to degrade lignocellulosic feedstock demonstrated the high potential in biomass and industrial application [18]. Also, a novel acid/ alkali and thermo tolerant cellulase with high ability in agro residues saccharification reported as a candidate for bioethanol production [19]. Many studies have focused on the isolation of novel endo- β-1,4-glucanases from metagenomic sources. For example, a novel halo-ionic liquids tolerant thermoacidophilic endo- β-1,4-glucanase was isolated from saline-alkaline lake soil microbial metagenomic DNA which could be applied in the hydrolysis of acid, and ionic liquid (IL) pretreated biomass [20]. Moreover, Song et al. isolated and characterized a new endo-β-1,4-glucanase from black-goat rumen metagenome [21] and Narra et al. mined a novel GH12 endo-glucanase which was applied in the enzymatic degradation of alkali-treated and delignified RS [22]. In another work Icelandic hot spring was used for isolation of a new halotolerant cellulase and used in biomass processing [23]. A new cellulase that showed thermal and halo stability, obtained from soil metagenome and reported as suitable candidate for various industrial application [24]. Direct cloning and using the metagenomics-guided strategy resulted in a thermostable and extremely salt tolerant cellulase for hydrolysis of the renewable biomass and biofuels production [25]. The rumen microbiota possesses a vigorous hydrolyzing enzyme profile adapted to enhance the digestion and exploitation of lignocellulosic biomass, which dominates the ruminant diet. In-silico screening provided the opportunity to explore the extensive biodiversity of nature and thus enabling the identification of several novel enzymes from different environments. Moreover, strong power of the metagenomic and in-silico screening in identification of the novel cellulases and hemicellulases with high ability to degrade lignocellulosic biomass was confirmed [26–29].

This work aimed to mine a novel thermostable alkaline endo-β-1,4- glucanases (PersiCel4) from the sheep rumen metagenomic data. After cloning, expression and purification of the enzyme, the characterization of the PersiCel4 was performed by measuring the optimal activity condition, and also the effect of different harsh condition such as presence of metal ions, different concentrations of salt, various temperatures and pH values on the enzymatic activity were analyzed. The PersiCel4 was then utilized to enhance the hydrolysis of pretreated RS and substantially increased the reducing sugar yield.

## Results

### Prediction of thermostable endo-glucanase sequences

The results obtained from the blast of six thermostable endo-glucanases mined from literature, against assembled contigs from rumen metagenome are presented in the supplementary S1. For each of the mentioned six enzymes, the 10 first results with the highest E-values are presented. The contigs with the E-values of lower than 1E-090 used for the next experiment.

One of the contigs assembled from the sheep rumen metagenome, named PersiCel4, passed all filters mentioned in the methods section, applied for identifying thermostable endo-glucanase. PersiCel4 was aligned against six characterized sequences obtained from literature (Table 1).

**Table 1:** The E-value, Identity percentage, and query coverage percentage for alignment between PersiCel4 and six thermostable endo-glucanase sequences obtained from literature mining

UniProt Accession	GH Family	EC	Organism	length	E-value	Identity %	Query Coverage %
P0C2S4	9	3.2.1.4	*Clostridium thermocellum* *(Ruminiclostridium thermocellum)*	625	0.74	25.76	19
Q60033	12	3.2.1.4	*Thermotoga maritima*	274	2.0	50.00	6
B7UAM4	5	3.2.1.4	*Bacillus subtilis*	499	3e-112	48.66	99
P54583	5	3.2.1.4	*Acidothermus cellulolyticus*	562	6e-04	21.72	57
P96492	12	3.2.1.4	*Thermotoga neapolitana*	274	1.1	36.36	6
G2QCS4.1	7	3.2.1.4	*Thermothelomyces thermophilus*	464	3.3	22.73	19

Also, the 100 most similar homologs of PersiCel4 were obtained from NCBI. Although none of them are thermostable cellulase, the E-value of the alignments for all homologs was less than 1E-104. The phylogenetic position of PersiCel4 among these mentioned characterized enzymes is demonstrated in the Supplementary S2 (A). Also, the phylogenetic tree that includes PersiCel4 and six characterized enzymes as well as 13 near homologs of PersiCel4 is demonstrated in the Supplementary S2 (B).

The PersiCel4 is an enzyme with amino acid length of 339. The blast results show that it is most similar to an endo-glucanase enzyme from GH5 family with Endohydrolysis of (1–4)-beta-D-glucosidic linkages in cellulose, lichenin and cereal beta-D-glucans catalytic activity and UniProt accession number B7UAM4 with E-Value of 3e-112 (Table 1).

**Fig. 1:**
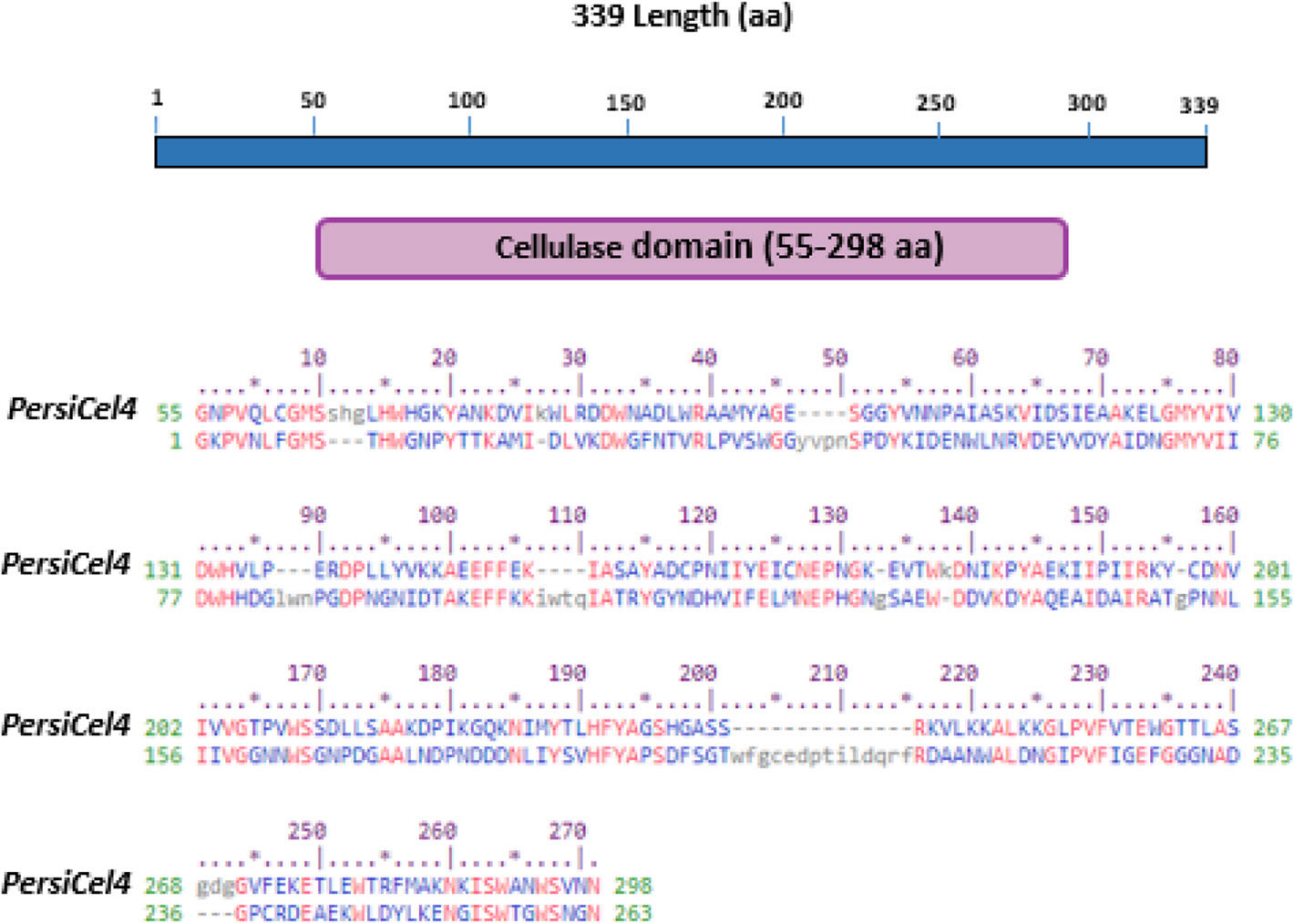
The PersiCel4 matched based on the CDD results

Based on the CDD results, the PersiCel4 best matches the Pssm-ID 333879 with Bit Score 212.21 and E-value 2.57e-67 (Fig. 1).

Phyre2 suggests that the most similar structure to the PersiCel4 with 100% confidence and 52% identity belongs to an endo-1,4-beta-glucanase from Bacillus subtilis 168 with 3PZV PDB code (Fig. 1).

### Expression and purification for recombinant endo-β-1,4-glucanase

After utilizing the DNA of the metagenomic source of sheep rumen, the over-expression of the degenerate primers was performed and the sequence of PersiCel4 fragment was amplified under the control of the T7 promoter of pET-28a vector in *E. coli* BL21 (DE3). To controlling the purification of the PersiCel4, the SDS-PAGE analysis was used and the single band of the PersiCel4 with a molecular weight of 38 kDa was showed in Supplementary S3.

### Properties of purified PersiCel4

**Fig. 2:**
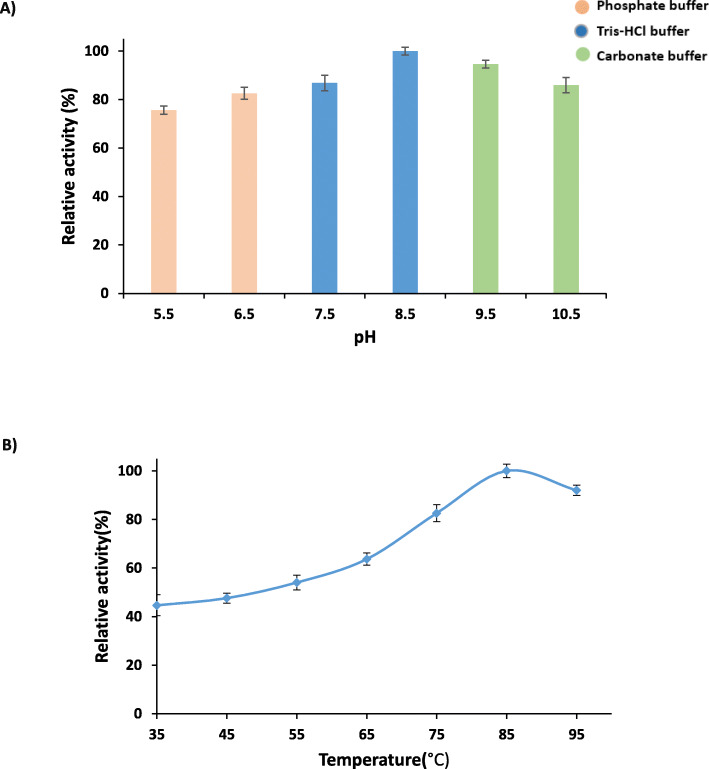
Effect of different pH values in various buffers (**a**) and different temperatures (**b**) on the activity of the PersiCel4

Optimum pH was determined in room temperature. Figure 2a shows that the optimal pH value for PersiCel4 was 8.5, and it preserved more than 75% of its maximum activity in the pH range between 5.5 up to 10.5. By fixing the pH for enzyme, the maximum activity was observed at 85 °C in the range of temperature between 30 and 90 °C, and the activity of the enzyme was decreased just 8% of its maximum value at 95 °C (Fig. 2b).

**Fig. 3:**
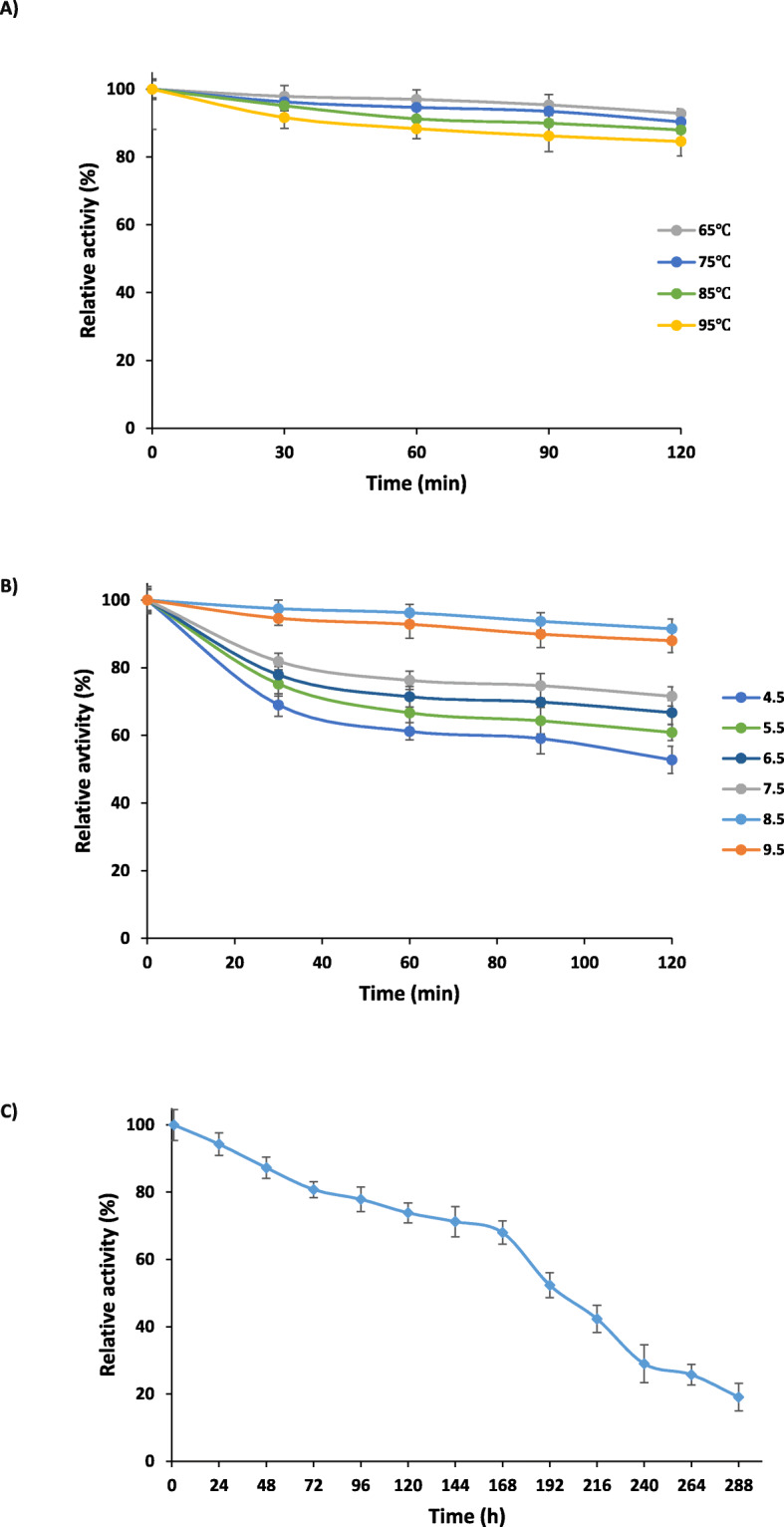
Thermal stability (**a**) of the enzyme after incubation for 30, 60, 90 and 120 min and pH stability (**b**) of the enzyme after incubation at pH 4.5, 5.5,6.5, 7.5, 8.5 and 9.5 (**c**) Storage stability of the PersiCel4 in 85 °C

The pH and thermal stability of the PersiCel4 were performed after incubation for 30-120 min. Based on the results from Fig. 3a the enzyme was greatly stable at high temperatures. It could retain 84, 87, 90 and 93% of its maximum activity after 120 min incubation at 95 °*C*, 85 °*C*, 75 °*C* and 65 °*C* respectively. The highest amounts of activities obtained at 65 °C and the enzyme exhibited the high stabilities at range of tested temperatures (65 °*C*-95 °*C*). As shown in Fig. 3b the PersiCel4 was stable at the pH values of analysis and demonstrated the maximum activities at alkali condition. The enzyme maintained the 88, 91 and 71% of its activities after 120 min incubation at pH 9.5, 8.5 and 7.5, correspondingly. The persiCel4 was also stable at lower pH values and showed 66, 61 and 53% activities at pH 6.5, 5.5 and 4.5.

Results of the kinetic studies showed that the PersiCel4 obeys the Michaelis-Menten kinetics. The K_m_ and V_max_ values of PersiCel4 were calculated 0.58 μmol ml^− 1^, 1.95 μmol min^− 1^ respectively. Also, the specific activity of the enzyme obtained 22.425 Umg^− 1^.

Storing the PersiCel4 for long period of time showed the stability of the enzyme and confirmed another trait of the purified endo-glucanase (Fig. 3c). The PersiCel4 retained 70% of its activity after 168 h at 85 °C and its activity decreased after 288 h of storage.

The PersiCel4 signified the stability and functionality at more extreme pH and thermal conditions when compared with other reported endo-β-1,4-glucanase.

The stability and activity of the PersiCel4 in broad range of pH, temperature and prolong storage and also the optimal condition at 85 °C and pH 8.5 which are higher than other endo-glucanase presented the capability of the PersiCel4 and its suitable characteristics among the many other reported endo-glucanases used for industrial waste application (Table 2).

**Table 2:** The property comparison of PersiCel4 with some known endo-β-1,4-glucanase representatives

Name	Source	Optimum pH	Optimum temperature	Proposed Application	References
**nmGH45**	saline-alkaline lake soil microbial metagenomic DNA	4.5	60–70	lignocellulosic biomass hydrolysis	(Zhao et al., 2018) [20, 30]
**CelG5**	Phialophora sp. G5	4.5	55–60	brewing and feed industries	(Zhao et al., 2012) [31]
**KG35**	metagenomic libraryof the black-goat rumen	6 … 7	30–50	ND^*^	(Song et al., 2017) [21]
**Cel5G**	metagenomic library from soil	4.8	50	industrial use	(Liu et al., 2011) [32]
**CS10**	metagenome gut microflora of *Hermetia illucens*	7	50	industrial use	(Lee et al., 2014) [33]
**cel5A**	mangrove soil	6.5–7.5	50	ND^*^	(Gao et al., 2010) [34]
**Cel5H**	*Dictyoglomus thermophilum*	5	50–85	ND^*^	(Shi et al., 2013) [35]
**CelRH5**	rhizosphere metagenomic library	6.5	40	ND^*^	(Wierzbicka-Woś et al., 2019) [36]
**Cel5R**	soil metagenome	5–6.5	58	ND^*^	(Garg et al., 2016) [24]
**cel7482, cel3623 and cel36**	Metagenomic sequences	5.5	60–70	ND^*^	(Yang et al., 2016) [25]
**Cel6H-f481**	compost metagenomic library	5.5	50	ND^*^	(Lee et al., 2018) [37]
PersiCel4	Sheep rumen metagenome	8.5	85	lignocellulosic biomass hydrolysis	This study

*ND Not Determined

### Effects of salts on enzyme activity

The PersiCel4 activity was investigated in the presence of different concentrations of chemicals in comparison with control in standard conditions (without any additive). According to the Table 3 the PersiCel4 was highly stable at the presence of several chemicals. Among the metal ions the maximum activity was obtained by MnCl_2_ (221.73%). Slight inhibition found by the CuSO_4_ (78.88%) and the enzyme maintained it is activity at the presence of other metal ions. Among the surfactants and inhibitors maximum activity obtained by the CTAB (145.03%) and negligible diminution observed by Urea (77.63%). Presence of the metal-chelating, EDTA displayed not observable effect on the enzymatic activity which showed that the enzyme could be a metal independence.

**Table 3:** PersiCel4’s relative activity (%) when treated with different concentrations of salts compared to control condition without the presence of salt

Metal ions	Relative activity (%)
Control	100
MgCl_2_	111.18
CaCl_2_	111.49
MnCl_2_	178.88
CuSO_4_	78.88
FeSO_4_	117.70
ZnCl_2_	118.01
Surfactants	**Relative activity (%)**
SDS	98.44
CTAB	145.03
Tween 20	107.76
Chemical modulators	**Relative activity (%)**
EDTA	96.58
Urea	77.63
PMSF	105.90
NaN_3_	81.67

**Fig. 4:**
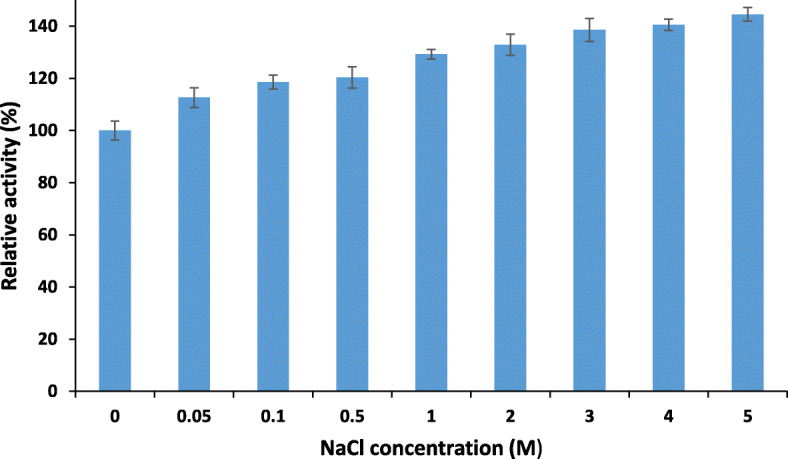
Effect of various concentrations of the NaCl on the PersiCel4 activity

Multiple concentrations of the NaCl was tested on the activity of the PersiCel4 and signified its salt tolerance Increment of the NaCl concentration from 0.05 M to 5 M demonstrated an elevation in the relative activities of the enzyme (Fig. 4). The PersiCel4 showed 144.51% of activity at the highest concentration of NaCl (5 M). This feature is yet to be added to other mentioned notable characteristics of PersiCel4 to nominate it for biofuel industries.

### Effect of different pretreatment and enzymatic hydrolysis of rice straw

In the pretreatment process, cellulose and hemicellulose can be removed, and cellulose crystallization reduced, and porosity of the materials increased use recent reference. An effective pretreatment method is the one which maximizes sugar productivity during the enzymatic hydrolysis step while preventing the degradation or loss of carbohydrates [12]. The effects of different preparations before enzymatic hydrolysis produced the highest sugar production.

**Fig. 5:**
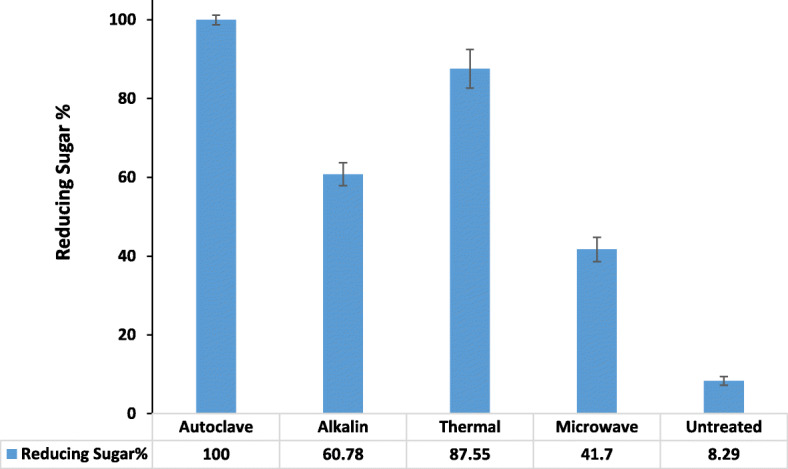
Comparing the generated reducing sugars of the RS after different pretreatment methods

Fig. 5 shows the percentage of reduced sugar in each pretreatment obtained from untreated and different treated RS. The highest yield of reducing sugar observed when the RS (2%w/v) in sodium phosphate buffer (50 mM, pH 7) had done with an incubator at 100 °C, 30 min by thermal pretreatment.

In this study, it observes that autoclave pretreatment RS improves enzymatic hydrolysis resulting in a higher yield of reducing sugar than untreated RS. This may be due to the deficiency caused by the swelling of RS particles and an increase in the inner surface, which in turn has led to access of more enzymes to the internal structure, and thereby, increasing sugar yields [38, 39].

**Fig. 6:**
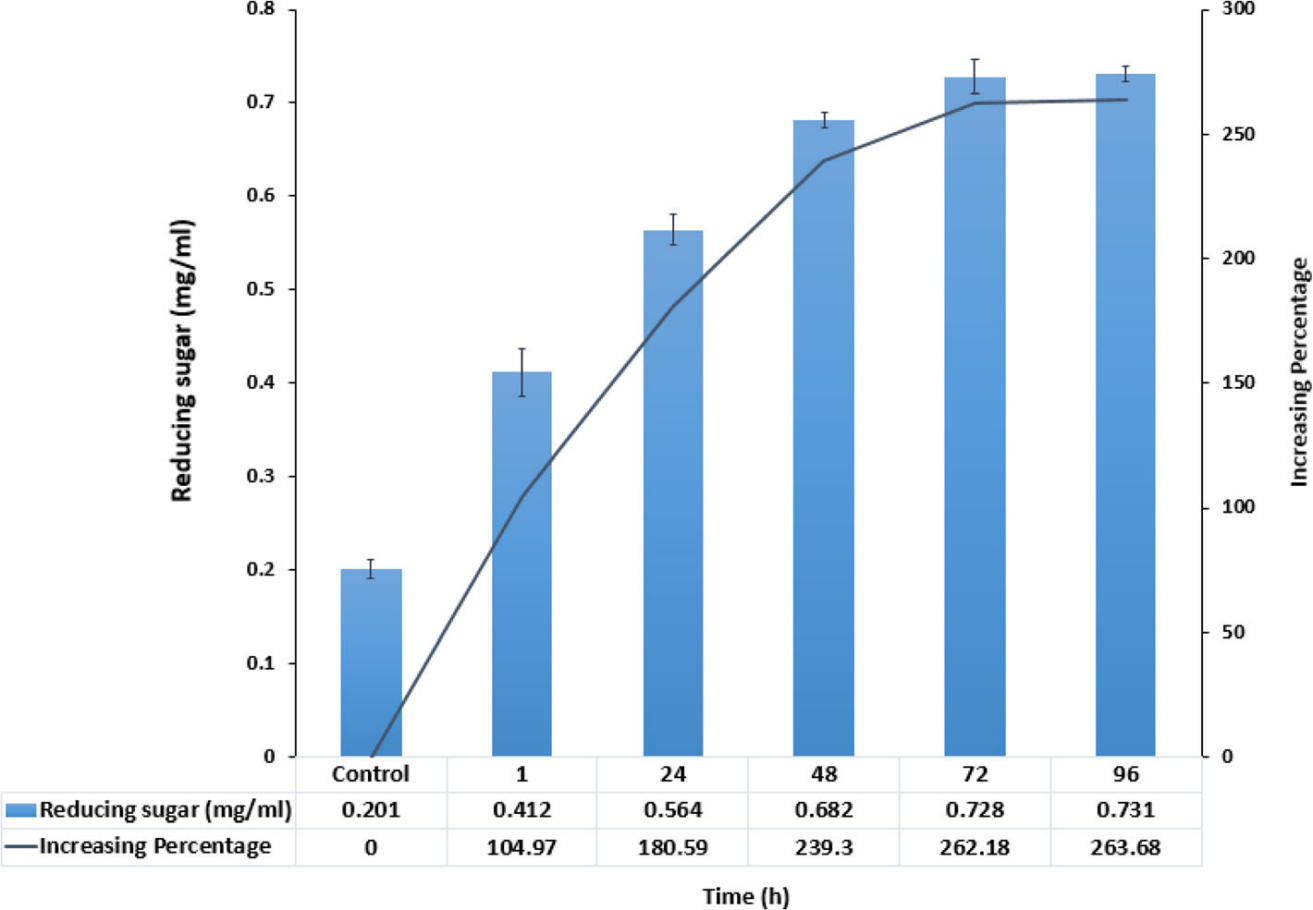
The amounts of the released reducing sugar in enzymatic hydrolysis of RS with PersiCel4 after 96 h

In the next step, the enzymatic hydrolysis of thermal treated RS was evaluated using screening of the reducing sugar concentrations against time, at 85 °C (pH 8.5) (Fig. 6).

The results showed that PersiCel4 significantly enhanced the saccharification yield in 96 h, specifically after 72 h, which confirmed the boosting effect PersiCel4. In autoclave treated RS, the comparison between enzymatic hydrolysis with the PersiCel4 and without it, revealed a 260% increase in the released reducing sugar after 72 h. In this study, it observes that the enzymatic hydrolysis of thermal pretreated RS was the most efficient method of converting the RS into reducing sugars. This study highlights the competence of the combined implementation of metagenomic screening and in-silico analysis in order to efficiently discover and isolate particular enzymes of interest. As described, PersiCel4 depicted various characteristics that are essential for enhanced hydrolysis of cellulose.

## Discussion

Based on the high potential of the metagenomic data for identifying new enzymes with valuable industrial applications, in current study a novel thermostable endo-glucanase was mined from the metagenomic data of sheep rumen using computational screening and named PersiCel4. After the in-silico screening, the enzyme cloned, expressed, purified. Characterization of the PersiCel4 demonstrated the optimal condition at temperature of 85 °C and pH 8.5. The PersiCel4 was stable at prolong storage and also was highly stable and active at high temperature and broad range of pH values.

The PersiCel4 showed considerable activity at the presence of various inhibitors and metal ions and could strongly maintain its activity at this condition. As described before, since several pretreatment methods take place at either acidic or alkaline conditions and due to formation of various salts at the pH neutralization step, an important challenge is the removal of such salts that consumes an abundance of water and energy [40]***.*** Given this fact, cellulolytic-enzymes that can tolerate the presence of different salts without reduction in their catalytic ability find attraction [9, 14]. Another property of the PersiCel4 was confirmed by the significant activity at the high concentration of NaCl (5 M) which showed the halo tolerant of the enzyme. The significant place of thermostability and halotolerant in the functionality of the novel endo-glucanases as the basic ingredients in lignocellulosic application was reported before [30]. In another study the stable endo-1,4-glucanase at the presence of metal ions and various reagents suggested as the promising candidate for utilization in biotechnological applications [41].

Lignocellulosic materials constitute economically renewable raw materials and are available in large quantities [42]. Several researchers have addressed the use of agricultural waste in the feasibility, sustainability, and economics of biofuels production [43–47].

Because of slow pace and incomplete hydrolysis of cellulose in moderate temperatures, the susceptibility of the microbial contamination on one hand and the easier recovery of ethanol at high temperatures and decreased importance of cooling after thermal pretreatment, on the other hand, thermostable enzymes find great importance and value [48, 49]. The competence of cellulases to operate in extreme pH conditions is another desirable attribute. Alkaline pretreatments on agricultural wastes such as RS have shown better sugar yield results compared to acid pretreatments [13] and alkali-stable cellulases have been reported to be suitable potential enzymes for lignocellulosic saccharification at such conditions [50].

One of the most abundant residues of lignocellulose in the world is RS [2, 51, 52]. Due to the complex structure of lignin and hemicelluloses, the conversion of RS to reducing sugar is a very complicated process [53]. Generally, by pretreatment of fibrous biomass through mechanical, physical, thermal, chemical or enzymatic pathways, it results in the conversion of complex cellulose and hemicellulose into reducing sugars. The effect of different pretreatment methods on the amounts of reducing sugars produced by RS demonstrated the maximum value of products with autoclaving. The enzymatic hydrolysis of treated RS indicated a significant increment in the saccharification yield after 96 h. In previous reports capacity of the novel endo-β-1,4-glucanase with a stimulatory effect on the production of fermented sugars was showed and reported as the suitable ingredient for the degradation of lignocellulosic biomass to biofuels [54]. Potency of the endo- β-1,4-glucanases in modification of biomass and agro residues such as RS for lignocellulosic saccharification and bioenergy industries was also confirmed before [55]. The alkali thermostable PersiCel4 with significant features including the activity and stability at long period of storage, wide range of pH and temperatures, halotolerant and activity in the existence of various chemical reagents proved its high performance on hydrolysis of RS and application in lignocellulosic biomass conversion.

## Conclusions

Waste management and energy crisis are among the most important issues that the world faces today. Such concerns have inspired the extensive search for capable novel enzymes to improve the recalcitrant lignocellulosic biomass conversion, such as agricultural wastes.

In this study, the sheep rumen metagenome was explored using in-silico analysis to find a novel alkali-thermostable halotolerant endo-β-1,4-glucanase. Ultimately, PersiCel4 was identified and isolated and characterized by multiple experiments, all of which verified the computationally predicted properties. PersiCel4 was successfully utilized for the hydrolysis of autoclave pretreated RS in extreme temperature and pH, which is the most plentiful agricultural waste. Regarding several proposed distinctive characteristics of PersiCel4, it can potentially find commercial applications the harsh conditions of the industrial bioconversion process.

## Methods

### Computational prediction of thermostable endo-β-1,4-glucanase enzyme sequences

The metagenomic library was prepared using the TruSeq DNA Library Preparation Kit (Illumina, San Diego, CA, USA), and the quantity assessment of the library was performed using a Qubit fluorimeter (Invitrogen, Carlsbad, CA, USA).

Raw sheep rumen metagenomic data was submitted to NCBI with Bioproject ID: PRJNA635543. The detailed information can be accessed in the related page at NCBI.

The primary and preprocessing operations, including quality control and short read assembly, carried out on sheep rumen metagenomics data using FastQC and MEGAHIT assembler respectively. After that, the assembled contigs were explored for putative thermostable endo-glucanase sequences by the filtering stages described below. In the first step, MetaGeneMark was employed for predicting potential microbial genes. The predicted endo-glucanase enzymes were separated from assembled contigs for next step analysis. Six thermostable endo-glucanase sequences from different GH families were identified from previous works and their sequences were downloaded from UniProt (Table 1). Using standalone NCBI BLAST, the mined enzymes from metagenome were aligned against the list of enzymes mined from literature. The most similar predicted metagenomics endo-glucanases to the known enzymes were determined based on pairwise blast with appropriate E-value.

Among predicted genes, those confirmed as endo-glucanases by NCBI Conserved Domains Database (CDD) [56] were selected for next step. The sequences of the filtered enzymes were assigned to the Phyre2 server [57] to predict their tertiary structures. Finally, one of the sequences that passed all filters, named PersiCel4, was selected for experimental assays.

### Cloning, expression, and purification of the endo-β-1,4-glucanase gene

Based on the metagenome DNA template of sheep rumen, the endo-β-1,4-glucanase gene encoding sequence was amplified by polymerase chain reaction (PCR) using PersiCel4 F (5′- TAATAGGCTAGCATGAAAAGAATTTTGATTTTGGC − 3′) and PersiCel4 R (5′- TGATAG GTCGAC TCACTCTATGTCTCCGCGA − 3′) primers which included *NdeI* and *NotI* restriction sites, respectively. PCR product was purified, digested with *NheI* and *SalI* and ligated into pET-28a, resulting recombinant plasmid pet28 [27, 28].

The agarose gel 1.5% (w/v) was utilized for recognition and purification of the PCR products. The DNA fragments digested into the pET28a and the resulting plasmids transformed into the *E. coli* BL21 (DE3). After the recombinant cultivation at 37 °*C*, the isopropyl-β-D-thiogalactopyranoside (IPTG) (0.4 mM) added and the enzyme expression induced at 20 °C for 20 h. The N-terminal Histidine-tagged recombinant protein was purified (Ni-NTA Fast Start Kit, Qiagen; Hilden, Germany) and approved with sodium dodecyl sulfate-polyacrylamide gel electrophoresis (SDS-PAGE).

After cloning, expression and purification, the enzyme named PersiCel4 and subjected to further biophysical experiments. Nucleotide sequence of PersiCel4 was submitted to Genbank Database with accession number Mn016942.

### Spectroscopy studies

Synergy™ HTX Multi-Mode Microplate Reader was used for UV–vis spectrophotometry. For matured protein concentration using the Bradford method [58], and bovine serum albumin (BSA) as the protein standard was used. The CMC substrate and reducing sugars amount was used for enzyme activity measurement by DNS method at 540 nm.

### Determination of optimum pH and temperature and storage

For determining the optimal pH of the PersiCel4, several buffers with different pH values including: phosphate buffer (50 mM, pH 5.5–6.5), Tris-HCl buffer (50 mM, pH 7.5–8.5), carbonate-bicarbonate buffer (50 mM pH 9.5–10.5) used. The enzyme added to the reaction mixture and incubated for 20 min at 25 °*C* followed by activity measurement based on the DNS assay [59]. To determine the optimum temperature of enzyme activity, the enzyme solution in 10 mM Tris-HCLbuffer (pH 8.5) with substrate was incubated in the different temperature (35–95 °C) for 20 min and the enzymatic activity was explored based on the DNS procedure.

For reporting PersiCel4 activity, relative activity was considered as the percentage of highest activity. The methods for determining storage stability was used by incubation of the purified enzymes for 12 days in kit elution buffer at 85 °C and the enzymes’ activities were examined in 24 h time intervals.

### Thermal and pH stability of PersiCel4

To analyzing the thermal stability, the enzyme was pre-incubated at different temperatures (65, 75, 85 and 95 °C) for 30, 60, 90 and 120 min. The pH stability was determined by pre-incubating the enzyme with various pH buffers including: Citrate sodium buffer (50 mM, pH 4.5–5.5), phosphate buffer (50 mM, pH 6.5–7.5), Tris-HCl buffer (50 mM, pH 8.5–9.5) at 85 °C for 30, 60, 90 and 120 min. DNS method was used to measuring the enzymatic activity. The relative activities were calculated respect to the 100% activity for control (0 h pre-incubation).

### Measurement of endo-β-1,4-glucanase activity and kinetic parameters

The cellulase activity was determined through the 3,5- dinitrosalicylic acid (DNS) assay [59] and using the carboxymethyl cellulose (CMC) as the substrate [60]. For this purpose, 20 μL of the PersiCel4 added to 60 μL of 0.5% solubilized CMC in Tris-HCL buffer (pH 8.5) and incubated at 85 °*C* for 20 min. After that, 120 μL of 3,5-dinitrosalicylic acid (DNS) reagent added to mixture to terminating the reaction and boiled for 5 min. The absorbance of the samples was measured at 540 nm via spectrophotometer.

The kinetic factors and specific activity of the PersiCel4 were determined using the substrate CMC. The one unit of the enzyme activity was defined as the amount of enzyme that releases 1 μmol of reducing sugar at a rate of 1 μmol per minute under the assay conditions, and the specific activity of the enzyme was determined as unit per mg of an enzyme in optimum condition (pH 8.5 and T 80 °C).

Ultimately, enzymes were incubated with CMC concentrations ranging from 0 to 10 mg/ml in 10 mM phosphate buffer at pH 8. Using Michaelis-Menten plots at room temperature, K_m_ and V_max_ and Michaelis constant were determined.

### Effect of metal ions and chemical reagents on the activity of PersiCel4

Different inhibitors including PMSF, EDTA, NaN_3_, Urea (5 mM), and SDS, CTAB, Tween 20 (1%) and several metal ions contains CaCl_2,_ MgCl_2_, CuSO_4_, MnCl_2_, FeSO_4_, ZnCl_2_ and NaCl (5 mM) were pe-incubated with the enzyme at 40 °C for 30 min without the substrate. The enzymatic activity was assayed according to the standard conditions. The enzyme activity without any chemicals was considered to be 100% activity and compared with the other values.

To analyzing the salt tolerant of the enzyme, different amounts of NaCl (0.05, 0.1, 0.5, 1, 2, 3, 4 and 5 M) in 50 mM phosphate buffer (pH 8.5) were used. The reaction mixtures were performed at optimum condition of the enzyme and their activities were measured according to the DNS assay.

### Rice straw hydrolysate (RSH)

Rice straw (RS) was collected from a local farm and washed with water and then dried at 70 °C in a hot air oven. By mixer grinder, size of dried RS has been reduced. The RS with suitable particle size (2–3 mm) pretreated by five different methods (alkaline, thermal, autoclave, microwave).

#### Alkaline pretreatments

In the RS (2%w/v), the pH was set at 12 by the addition of 2 M NaOH, then mixed with magnetic stirrer for 30 min [61].

#### Autoclave pretreatments

The RS (2%w/v) in sodium phosphate buffer (50 mM, pH 7) have been done with autoclave at 121 °C, 1.5 atm for 15 min [62].

#### The microwave pretreatments

About 0.2 g of RS was suspended in 10 mL of sodium phosphate buffer (50 mM, pH 7) at 30 min in microwave. For microwave pretreatment 700 W; 170 °C; 15 min were chosen [63].

#### Thermal pretreatments

For thermal pretreatment, the RS (2%w/v) in sodium phosphate buffer (50 mM, pH 7) have been done with an incubator at 100 °C, 30 min [64].

To finding the best pretreatment with highest effectiveness, the amounts of released reducing sugars were determined. For this goal, after the pretreatment, the substrates were subjected to distillation and centrifuged for several times. Then, the samples were dried at 50 °C overnight and stored in a cool and dry place for further analysis. In the next step, the pre-treated RS was suspended in 50 mM sodium phosphate buffer (pH 8.5) followed by the enzyme addition. By using the incubator shaker at 120 rpm at 85 °C, the substrates were saccharified for 96 h. After the centrifugation, the generated reducing sugars were measured by DNS assay.

## Supplementary information

**Additional file 1.**

**Additional file 2.**

**Additional file 3.**

## Data Availability

The Sheep rumen metagenomic data can be accessed from NCBI with Bioproject ID PRJNA635543. The detailed information can be accessed in the related page at NCBI. The PersiCel4 sequence is accessible from NCBI with Mn016942 accession number.

## Abbreviations

GH: Glycoside hydrolase
IL: Ionic liquid
CDD: Conserved domains database
PCR: Polymerase chain reaction
IPTG: Isopropyl-β-D-thiogalactopyranoside
SDS-PAGE: Dodecyl sulfate-polyacrylamide gel electrophoresis
BSA: Bovine serum albumin
OD: Optical density
DNS: Nitro of 3,5- dinitrosalicylic acid
CMC: Carboxymethyl cellulose
RS: Rice straw
RSH: Rice straw hydrolysate
