# Supplementary material for: A novel high performance in-silico screened metagenome-derived alkali-thermostable endo-β-1,4-glucanase for lignocellulosic biomass hydrolysis in the harsh conditions
Source: BMC Biotechnol. 2020 Oct 19;20:56. doi: 10.1186/s12896-020-00647-6 (PMC7574624; doi:10.1186/s12896-020-00647-6)
Supplement: Supplementary file 1 — Additional file 1. [file 12896_2020_647_MOESM1_ESM.docx]

**Supplementary S1:**

**Results obtained from the blast of six thermostable endo-glucanases mined from literature, against assembled contigs from rumen metagenome are presented here. For each of the mentioned six enzymes, the 10 first results with the highest E-values are presented. All contigs with E-values less than 1E-090 were selected for further analysis.**

**The assembled contigs will be available upon request.**

1. **Query= P0C2S4.1**|GUND_CLOTM RecName: Full=Endoglucanase D; Short=EGD;

AltName: Full=Cellulase D; AltName: Full=Endo-1,4-beta-glucanase;

Flags: Precursor

Length=625

Score E

Sequences producing significant alignments: (Bits) Value

scaffold_1269_33 # 35944 # 38256 # -1 # ID=1270_33;partial=00;s... 296 2e-091

scaffold_1601_15 # 15702 # 18941 # -1 # ID=1602_15;partial=00;s... 298 1e-089

scaffold_2175_28 # 29511 # 31823 # 1 # ID=2176_28;partial=00;st... 278 2e-084

scaffold_2055_29 # 36564 # 38810 # 1 # ID=2056_29;partial=00;st... 275 2e-083

scaffold_2086_6 # 5125 # 7488 # 1 # ID=2087_6;partial=00;start_... 268 1e-080

scaffold_1081_74 # 85846 # 87552 # -1 # ID=1082_74;partial=00;s... 262 3e-080

scaffold_2050_21 # 24219 # 25970 # 1 # ID=2051_21;partial=00;st... 262 3e-080

scaffold_502_36 # 38673 # 41003 # 1 # ID=503_36;partial=00;star... 262 1e-078

scaffold_2416_21 # 29200 # 30990 # 1 # ID=2417_21;partial=00;st... 252 3e-076

scaffold_1404_6 # 6608 # 8170 # 1 # ID=1405_6;partial=00;start_... 239 4e-072

1. **Query= Q60033** | CAA93274.1 endo-1,4-beta-glucanase [Thermotoga maritima MSB8]

Length=274

Score E

Sequences producing significant alignments: (Bits) Value

scaffold_1171_35 # 33416 # 35203 # -1 # ID=1172_35;partial=00;s... 28.5 0.99

scaffold_2149_31 # 47055 # 48482 # -1 # ID=2150_31;partial=00;s... 27.3 2.0

scaffold_2583_8 # 12205 # 14400 # 1 # ID=2584_8;partial=00;star... 26.9 2.8

scaffold_185_69 # 77683 # 80682 # 1 # ID=186_69;partial=00;star... 26.6 3.3

scaffold_653_41 # 54458 # 55147 # -1 # ID=654_41;partial=00;sta... 26.2 4.1

scaffold_969_3 # 1588 # 2460 # 1 # ID=970_3;partial=00;start_ty... 26.2 4.5

scaffold_2672_4 # 9544 # 11889 # 1 # ID=2673_4;partial=00;start... 26.2 4.9

scaffold_1273_18 # 16874 # 18073 # 1 # ID=1274_18;partial=00;st... 25.8 6.0

scaffold_1270_34 # 35707 # 37332 # 1 # ID=1271_34;partial=00;st... 25.8 6.7

1. **Query= B7UAM4** | ACK38261.1 endo-1,4-beta-glucanase [Bacillus subtilis]

Length=499

Score E

Sequences producing significant alignments: (Bits) Value

**This enzyme named PersiCel4:**

scaffold_2105_8 # 7628 # 8647 # -1 # ID=2106_8;partial=00;start... 325 3e-109

scaffold_591_65 # 81447 # 82463 # -1 # ID=592_65;partial=00;sta... 307 6e-102

scaffold_988_33 # 34002 # 35663 # 1 # ID=989_33;partial=00;star... 313 2e-101

scaffold_800_30 # 37809 # 38999 # 1 # ID=801_30;partial=00;star... 304 4e-100

scaffold_291_43 # 51321 # 52415 # 1 # ID=292_43;partial=00;star... 300 4e-099

scaffold_212_73 # 73092 # 74876 # 1 # ID=213_73;partial=00;star... 306 2e-098

scaffold_590_12 # 15675 # 17534 # 1 # ID=591_12;partial=00;star... 306 4e-098

scaffold_609_31 # 35603 # 36934 # 1 # ID=610_31;partial=00;star... 295 9e-096

scaffold_634_66 # 77702 # 79312 # 1 # ID=635_66;partial=00;star... 296 4e-095

scaffold_2053_26 # 31216 # 32772 # -1 # ID=2054_26;partial=00;s... 294 1e-094

1. **Query= P54583** | sp|P54583.1|GUN1_ACIC1 RecName: Full=Endoglucanase E1; AltName:

Full=Cellulase E1; AltName: Full=Endo-1,4-beta-glucanase E1;

AltName: Full=Endocellulase E1; Flags: Precursor

Length=562

Score E

Sequences producing significant alignments: (Bits) Value

scaffold_1012_64 # 73650 # 75341 # 1 # ID=1013_64;partial=00;st... 250 1e-076

scaffold_1676_7 # 7935 # 10007 # 1 # ID=1677_7;partial=00;start... 228 2e-067

scaffold_988_34 # 35697 # 37334 # 1 # ID=989_34;partial=00;star... 224 4e-067

scaffold_447_98 # 102224 # 104239 # -1 # ID=448_98;partial=00;s... 226 9e-067

scaffold_1014_43 # 46750 # 48837 # 1 # ID=1015_43;partial=00;st... 222 5e-065

scaffold_466_2 # 926 # 3094 # 1 # ID=467_2;partial=00;start_typ... 206 5e-059

scaffold_2510_38 # 35723 # 37480 # -1 # ID=2511_38;partial=00;s... 192 9e-055

scaffold_683_43 # 56699 # 58765 # 1 # ID=684_43;partial=00;star... 60.1 4e-010

scaffold_988_33 # 34002 # 35663 # 1 # ID=989_33;partial=00;star... 55.1 1e-008

scaffold_1000_13 # 16316 # 17557 # 1 # ID=1001_13;partial=00;st... 50.4 3e-007

1. **Query= P96492** | AAC95060.1 endo-1,4-beta-glucanase B [Thermotoga neapolitana]

Length=274

Score E

Sequences producing significant alignments: (Bits) Value

scaffold_969_3 # 1588 # 2460 # 1 # ID=970_3;partial=00;start_ty... 29.3 0.40

scaffold_1171_35 # 33416 # 35203 # -1 # ID=1172_35;partial=00;s... 28.5 0.77

scaffold_1572_31 # 31661 # 32521 # -1 # ID=1573_31;partial=00;s... 27.7 1.3

scaffold_903_23 # 24637 # 27024 # -1 # ID=904_23;partial=00;sta... 26.6 3.6

scaffold_1273_18 # 16874 # 18073 # 1 # ID=1274_18;partial=00;st... 26.2 4.7

scaffold_269_47 # 47381 # 49843 # 1 # ID=270_47;partial=00;star... 26.2 5.0

scaffold_1407_39 # 50993 # 53491 # -1 # ID=1408_39;partial=00;s... 26.2 5.4

scaffold_1270_34 # 35707 # 37332 # 1 # ID=1271_34;partial=00;st... 25.8 5.8

scaffold_377_38 # 45679 # 46911 # -1 # ID=378_38;partial=00;sta... 25.8 6.2

scaffold_2149_31 # 47055 # 48482 # -1 # ID=2150_31;partial=00;s... 25.4 7.7

1. **Query= G2QCS4.1**|CEL7A_MYCTT RecName: Full=Endoglucanase 7a; AltName:

Full=Cellulase 7a; AltName: Full=Endo-1,4-beta-glucanase 7a; Flags:

Precursor

Length=464

Score E

Sequences producing significant alignments: (Bits) Value

scaffold_2044_56 # 57691 # 60024 # -1 # ID=2045_56;partial=00;s... 30.8 0.32

scaffold_557_12 # 22694 # 25120 # -1 # ID=558_12;partial=00;sta... 28.9 1.5

scaffold_1569_29 # 35871 # 37685 # -1 # ID=1570_29;partial=00;s... 26.9 4.8

scaffold_901_47 # 59537 # 61624 # 1 # ID=902_47;partial=00;star... 26.6 7.0

scaffold_889_17 # 18803 # 20116 # -1 # ID=890_17;partial=00;sta... 26.2 8.5
