# Supplementary material for: A novel high performance in-silico screened metagenome-derived alkali-thermostable endo-β-1,4-glucanase for lignocellulosic biomass hydrolysis in the harsh conditions
Source: BMC Biotechnol. 2020 Oct 19;20:56. doi: 10.1186/s12896-020-00647-6 (PMC7574624; doi:10.1186/s12896-020-00647-6)
Supplement: Supplementary file 2 — Additional file 2. [file 12896_2020_647_MOESM2_ESM.docx]

**Supplementary S2:**

**100 most similar homologs of PersiCel4 were obtained from NCBI. Although none of them are thermostable cellulase, the E-value of the alignments for all homologs was less than 1E-104. The phylogenetic position of PersiCel4 among these six mentioned characterized enzymes is demonstrated in the Supplementary S2 (A). Also, the phylogenetic tree that includes PersiCel4 and six characterized enzymes as well as 13 near homologs of PersiCel4 is demonstrated in the Supplementary S2 (B).**

**The PersiCel4 and other characterized thermostable cellulases are marked with red rectangles. The Figure shows that PersiCel4 with all thermostable enzymes (other than B7UAM4) are in two different clades. It is clear that the sequence similarity alone, is not sufficient for identification of enzymes with desired properties.**


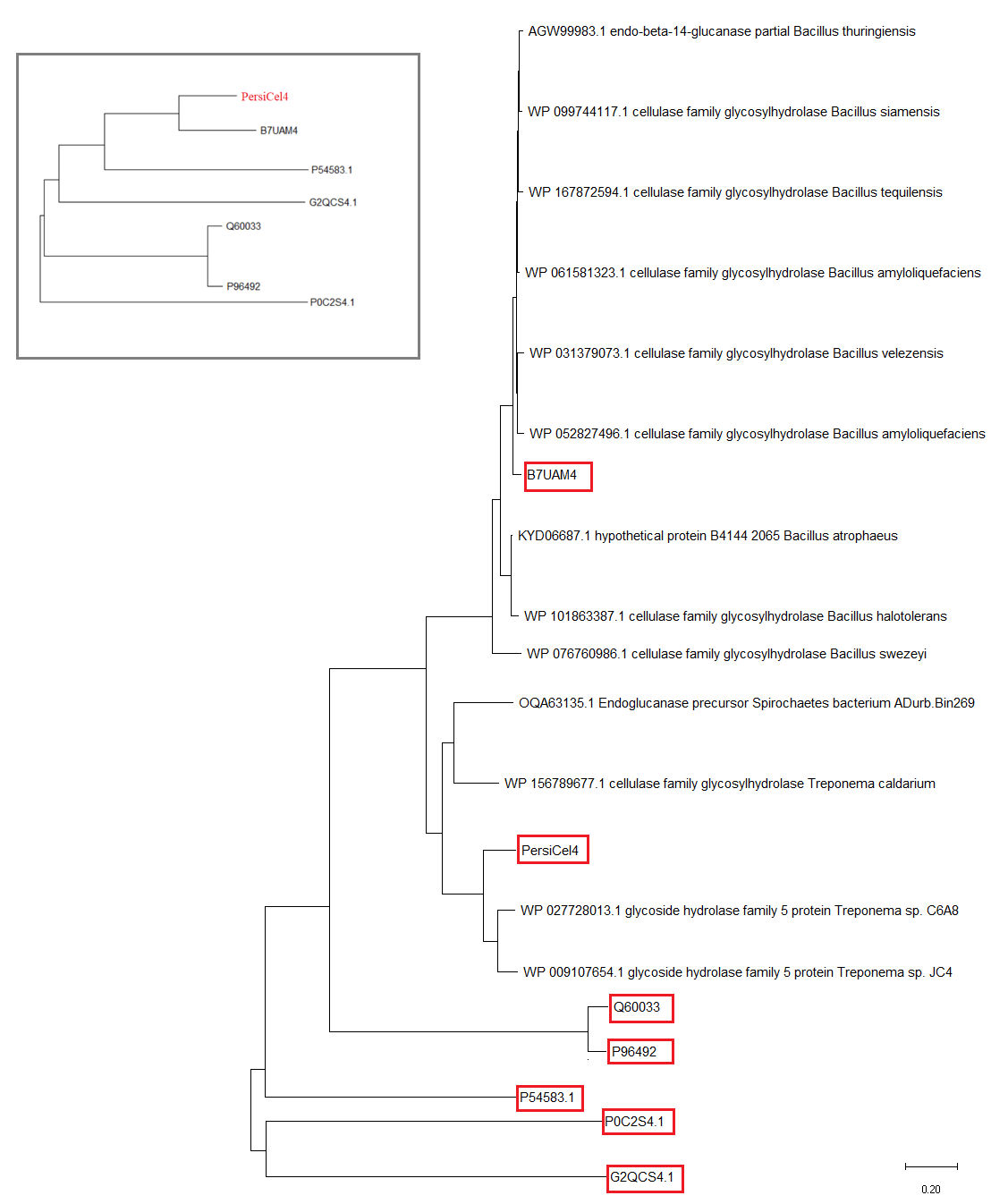


**A)**

**B)**

**The evolutionary history was inferred using the Neighbor-Joining method [1]. The optimal tree with the sum of branch length = 6.05835518 is shown. The tree is drawn to scale, with branch lengths in the same units as those of the evolutionary distances used to infer the phylogenetic tree. The evolutionary distances were computed using the Poisson correction method [2] and are in the units of the number of amino acid substitutions per site. This analysis involved 7 amino acid sequences. All ambiguous positions were removed for each sequence pair (pairwise deletion option). There were a total of 648 positions in the final dataset. Evolutionary analyses were conducted in MEGA X [3].**

1. Saitou N. and Nei M. (**1987**). The neighbor-joining method: A new method for reconstructing phylogenetic trees. *Molecular Biology and Evolution* **4**:406-425.

2. Zuckerkandl E. and Pauling L. (**1965**). Evolutionary divergence and convergence in proteins. Edited in *Evolving Genes and Proteins* by V. Bryson and H.J. Vogel, pp. 97-166. Academic Press, New York.

3. Kumar S., Stecher G., Li M., Knyaz C., and Tamura K. (**2018**). MEGA X: Molecular Evolutionary Genetics Analysis across computing platforms. *Molecular Biology and Evolution* **35**:1547-1549.
