# Supplementary material for: A novel high performance in-silico screened metagenome-derived alkali-thermostable endo-β-1,4-glucanase for lignocellulosic biomass hydrolysis in the harsh conditions
Source: BMC Biotechnol. 2020 Oct 19;20:56. doi: 10.1186/s12896-020-00647-6 (PMC7574624; doi:10.1186/s12896-020-00647-6)
Supplement: Supplementary file 3 — Additional file 3. [file 12896_2020_647_MOESM3_ESM.docx]

**Supplementary S3:**


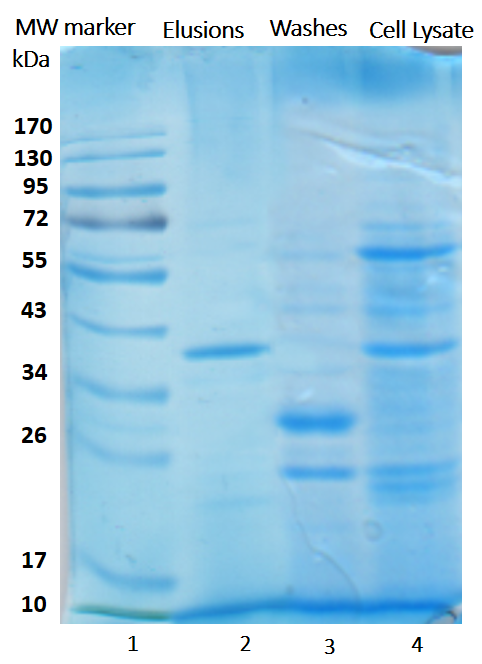


**SDS-PAGE analysis of** **recombinant endo-β-1,4-glucanase. SDS-PAGE was performed using a 12 % polyacrylamide gel and stained with Coomassie Brilliant Blue. Lane 1 molecular weight of marker standard. . Lane 2 purified recombinant endo-β-1,4-glucanase. Lane 4 shows** **supernatant of *E.coli’s* lysis containing recombinant endo-β-1,4-glucanase.**

**
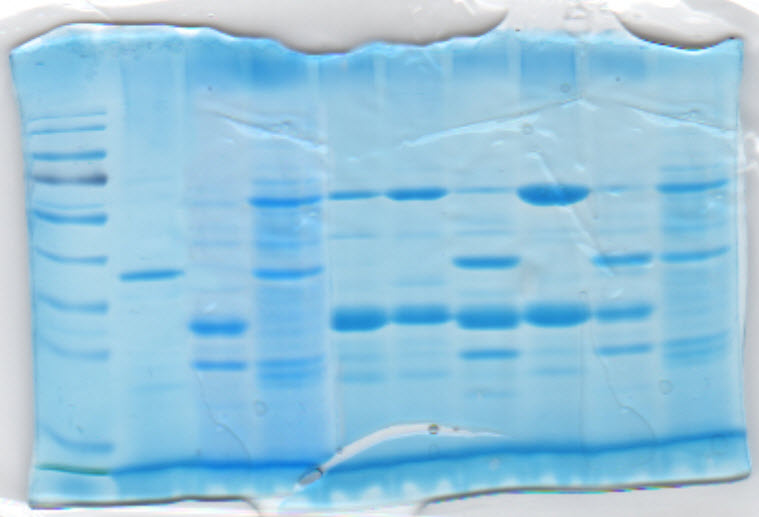
**

**The original full length uncropped version of the SDS-page gel.**


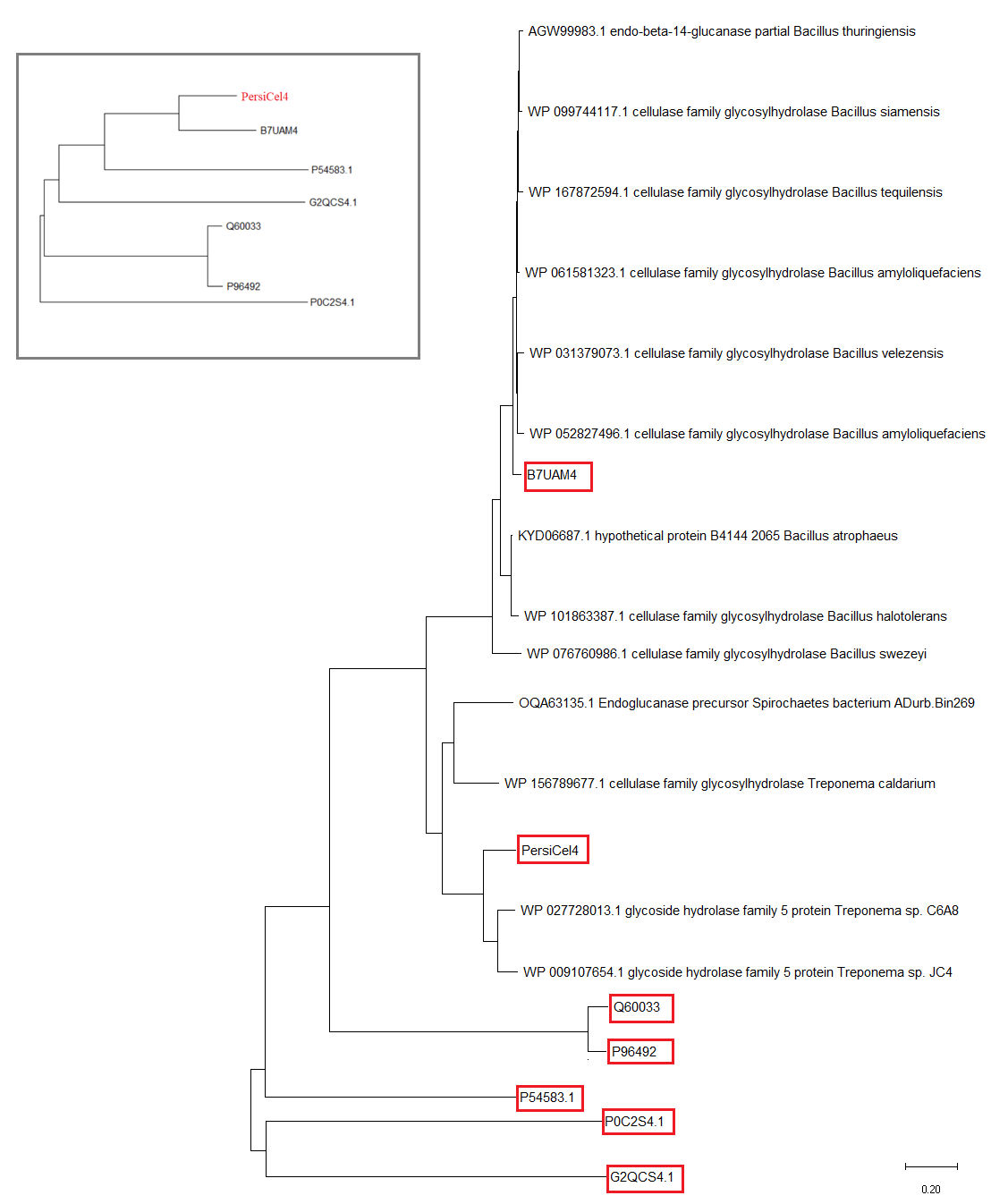


**A)**

**B)**

**The evolutionary history was inferred using the Neighbor-Joining method [1]. The optimal tree with the sum of branch length = 6.05835518 is shown. The tree is drawn to scale, with branch lengths in the same units as those of the evolutionary distances used to infer the phylogenetic tree. The evolutionary distances were computed using the Poisson correction method [2] and are in the units of the number of amino acid substitutions per site. This analysis involved 7 amino acid sequences. All ambiguous positions were removed for each sequence pair (pairwise deletion option). There were a total of 648 positions in the final dataset. Evolutionary analyses were conducted in MEGA X [3].**

1. Saitou N. and Nei M. (**1987**). The neighbor-joining method: A new method for reconstructing phylogenetic trees. *Molecular Biology and Evolution* **4**:406-425.

2. Zuckerkandl E. and Pauling L. (**1965**). Evolutionary divergence and convergence in proteins. Edited in *Evolving Genes and Proteins* by V. Bryson and H.J. Vogel, pp. 97-166. Academic Press, New York.

3. Kumar S., Stecher G., Li M., Knyaz C., and Tamura K. (**2018**). MEGA X: Molecular Evolutionary Genetics Analysis across computing platforms. *Molecular Biology and Evolution* **35**:1547-1549.
